# Estimation of kinetic parameters related to biochemical interactions between hydrogen peroxide and signal transduction proteins

**DOI:** 10.3389/fchem.2014.00082

**Published:** 2014-10-02

**Authors:** Paula M. Brito, Fernando Antunes

**Affiliations:** ^1^URIA-Centro de Patogénese Molecular, Faculdade de Farmácia, Universidade de LisboaLisboa, Portugal; ^2^Instituto de Medicina Molecular, Faculdade de Medicina da Universidade de LisboaLisboa, Portugal; ^3^Faculdade de Ciências da Saúde, Universidade da Beira InteriorCovilhã, Portugal; ^4^Departamento de Química e Bioquímica and Centro de Química e Bioquímica, Faculdade de Ciências, Universidade de LisboaLisboa, Portugal

**Keywords:** redox regulation, redox signaling, kinetics, rate constant, PTP1B, SHP-2, protein tyrosine phosphatases

## Abstract

The lack of kinetic data concerning the biological effects of reactive oxygen species is slowing down the development of the field of redox signaling. Herein, we deduced and applied equations to estimate kinetic parameters from typical redox signaling experiments. H_2_O_2_-sensing mediated by the oxidation of a protein target and the switch-off of this sensor, by being converted back to its reduced form, are the two processes for which kinetic parameters are determined. The experimental data required to apply the equations deduced is the fraction of the H_2_O_2_ sensor protein in the reduced or in the oxidized state measured in intact cells or living tissues after exposure to either endogenous or added H_2_O_2_. Either non-linear fittings that do not need transformation of the experimental data or linearized plots in which deviations from the equations are easily observed can be used. The equations were shown to be valid by fitting to them virtual time courses simulated with a kinetic model. The good agreement between the kinetic parameters estimated in these fittings and those used to simulate the virtual time courses supported the accuracy of the kinetic equations deduced. Finally, equations were successfully tested with real data taken from published experiments that describe redox signaling mediated by the oxidation of two protein tyrosine phosphatases, PTP1B and SHP-2, which are two of the few H_2_O_2_-sensing proteins with known kinetic parameters. Whereas for PTP1B estimated kinetic parameters fitted in general the present knowledge, for SHP-2 results obtained suggest that reactivity toward H_2_O_2_ as well as the rate of SHP-2 regeneration back to its reduced form are higher than previously thought. In conclusion, valuable quantitative kinetic data can be estimated from typical redox signaling experiments, thus improving our understanding about the complex processes that underlie the interplay between oxidative stress and redox signaling responses.

## Introduction

Being higher reductions states of molecular dioxygen, reactive oxygen species are present in all aerobic organisms. Initially, these species were seen as harmful species that caused or participated in the etiology of many diseases through oxidative damage, but more recently physiological roles mediated by the modulation of the redox state of biomolecules were attributed to reactive oxygen species (Sies, 2014). Today redox biology is an established field. As Berzelius put it the venom is in the dose, and reactive oxygen species have different roles depending on their concentration. This work is centered on hydrogen peroxide (H_2_O_2_), a reactive oxygen species that has the properties of a second messenger (Forman et al., 2010) and participates in many pathways, including insulin (Mahadev et al., 2001; Haque et al., 2011), mitogenic (Irani et al., 1997), inflammatory, and apoptotic signaling (Oakley et al., 2009; Tschopp and Schroder, 2010). Having a relative low chemical reactivity, H_2_O_2_ reacts mainly with metal centers and with thiol compounds, such as cysteine residues in proteins (Marinho et al., 2014). Examples of H_2_O_2_ targets are PerR, a metal-dependent transcription factor that is inhibited by H_2_O_2_ in a Fenton-like reaction, and protein tyrosine phosphatases (PTPs), which are inhibited upon oxidation of cysteine residues in their active center (Tanner et al., 2011; Marinho et al., 2014; Sies, 2014). The list of proteins containing cysteine residues that were observed to be oxidized by H_2_O_2_ is vast, near 200 (Le Moan et al., 2006; Martínez-Acedo et al., 2012), and continues to increase as investigators find new targets for H_2_O_2_. In contrast, kinetic parameters concerning oxidation by H_2_O_2_ have been measured only for a few of these proteins (Ferrer-Sueta et al., 2011; Tanner et al., 2011), leading several researchers to point out the lack of proper quantitative data as a barrier to the development of the field (Brigelius-Flohé and Flohé, 2011; Buettner et al., 2013). Importantly, the triggering of biphasic responses by H_2_O_2_ in a narrow concentration range has important biological implications. For example, in H4IIEC hepatocytes H_2_O_2_ can either enhance or impair insulin signaling depending on its concentration (Iwakami et al., 2011). This dual role was attributed to the different sensitivity of PTP1B inhibition and JNK activation, two kinases that stimulate and inhibit insulin signaling, respectively. Thus, while H_2_O_2_ is an essential component of the insulin signaling pathway, it may also mediate the etiology of insulin resistance (Fisher-Wellman and Neufer, 2012). Although the underlying data is known for some time, such picture only emerged recently, probably because in absence of a quantitative framework, these biphasic responses were often interpreted as contradictory findings that were dependent of the biological model used or simply reflected non-reproducible experimental results. To study such complex responses it is advantageous to apply a quantitative and integrative approach typical of systems biology (Buettner et al., 2013), where the reactivity of targets toward H_2_O_2_ is determined to undercover which pathways operate *in vivo* under different conditions. In this work, we address how kinetic parameters can be determined from typical experiments performed in redox signaling.

Based on a simple reaction scheme representing H_2_O_2_ signaling, we started by deducing kinetic equations that are tailored to estimate kinetic parameters from experimental data. Next, to test the validity of the deduced equations, virtual experiments carried out under different conditions of H_2_O_2_ exposure were simulated with a kinetic model, and the results were fitted to the equations deduced. The agreement between the kinetic parameters obtained in these fittings and those used to obtain the virtual time courses was used as a criterion to decide on the accuracy of the kinetic equations deduced. Finally, to evaluate the applicability of kinetic equations to real data, experimental results described in the literature focusing on PTP-dependent signaling were fitted to the equations deduced. Two PTPs, PTP1B, and SHP-2, for which kinetic rate constants are known, were chosen as test cases. Our study demonstrates that insightful kinetic parameters related to biochemical interactions between H_2_O_2_ and signal transduction proteins can be estimated from typical H_2_O_2_-signaling experiments by applying the equations deduced here.

## Theory and methods

### Master equation

A minimal mathematical analytical model was set up to describe a signaling event triggered by H_2_O_2_, according to the following two reactions:

(1) $${\text{Target}}_{\text{rd}}+{\text{H}}_{2}{\text{O}}_{2}\to {\text{Target}}_{\text{ox}}+{\text{H}}_{2}\text{O}$$

(2) $${\text{Target}}_{\text{ox}}\to {\text{Target}}_{\text{rd}}$$

In the first reaction, the reduced form of a sensor protein target (Target_rd_) is oxidized by H_2_O_2_, modifying its activity, which results in the modulation of a signaling pathway. In the second reaction, the oxidized target (Target_ox_) is switched-off by being regenerated back to the reduced form. A specific example of these two reactions is the inhibition of PTPs by oxidation of cysteine residues in their active center, which are reactivated upon reduction of this site; the temporary inhibition of these phosphatases increases the level of phosphorylation of their targets, thus promoting the signaling process. For these two reactions rate laws were defined as follows:

- For the H_2_O_2_-dependent oxidation step (1) *v*_1_ = *k_activation_* × [Target_rd_], where *k_activation_* = *k_target + H2O2_* × [H_2_O_2_]. *k_target + H2O2_* is the rate constant for the direct oxidation of the target protein by H_2_O_2_.
- For the switch-off step (2), *v*_2_ = *k_switchoff_* × [Target_ox_]. The total concentration of the target protein is assumed to be constant within the duration of the experiment ([Target]_total_ = [Target_ox_] + [Target_rd_]), and so *v*_2_ = *k_switchoff_* × ([Target]_total_ − [Target_*rd*_]).

Based on these two chemical reactions, the following differential equation was set up, where Target_rd_ is the fraction of the target protein in the reduced state, *t* is time, and *d/dt* stands for the differential operator:

(3) $$\frac{d{\text{Target}}_{\text{rd}}}{dt}=k_{switchoff}\left(1-{\text{Target}}_{\text{rd}}\right)-k_{activation}{\text{Target}}_{\text{rd}}$$

The master equation describing the time course of Target_rd_ is given by the analytical solution of Equation (3):

(4) $$\begin{array}{l} {{\text{Target}}_{\text{rd}}|}_{t}=\frac{k_{switchoff}}{k_{switchoff}+k_{activation}}+e^{-\left(k_{switchoff}+k_{activation}\right)\times t}\\ \;\times \left({{\text{Target}}_{\text{rd}}|}_{0}-\frac{k_{switchoff}}{k_{switchoff}+k_{activation}}\right) \end{array}$$

With

(5) $${{\text{Target}}_{\text{ox}}|}_{t}={1-{\text{Target}}_{\text{rd}}|}_{t}$$

Target_rd_ |_*t*_ and Target_ox_ |_*t*_ are the fractions of the target protein in the reduced and oxidized state at time *t*, respectively. Once the experimental variation of these fractions with time is known and the fraction of reduced target at time 0 (Target_ox_ |_0_) is measured, a non-linear fit to Equation (4) can be applied to estimate the kinetic parameters *k_activation_* and *k_switchoff_*. One possibility is to estimate these two unknown parameters from a two-parameter non-linear fitting. Alternatively, if one of these parameters is already known, only the remaining unknown parameter is estimated from a one-parameter non-linear fitting.

Next, we linearized Equation (4) so that kinetic parameters can be determined from linear plots, in which deviations from the master Equation (4) are easier to observe. To linearize Equation (4), the steady-state (ss) fraction of protein present in the reduced form, Target_ox_ |_ss_, was obtained by letting *t* to tend to infinite, resulting in Equation (6):

(6) $${{\text{Target}}_{\text{rd}}|}_{ss}=\frac{k_{switchoff}}{k_{switchoff}+k_{activation}}$$

Equation (6) was used to rewrite and linearize Equation (4) as Equations (7) and (8). If in the experimental time course this steady-state is not observed, Equation (4) cannot be linearized according to this procedure.

(7) $$\frac{{{\text{Target}}_{\text{rd}}|}_{t}-{{\text{Target}}_{\text{rd}}|}_{ss}}{{{\text{Target}}_{\text{rd}}|}_{0}-{{\text{Target}}_{\text{rd}}|}_{ss}}=e^{-\left(k_{switchoff}+k_{activation}\right)\times t}$$

(8) $$\text{ln}\left(\frac{{{\text{Target}}_{\text{rd}}|}_{t}-{{\text{Target}}_{\text{rd}}|}_{ss}}{{{\text{Target}}_{\text{rd}}|}_{0}-{{\text{Target}}_{\text{rd}}|}_{ss}}\right)\text{​}=\text{​}-\left(k_{switchoff}+k_{activation}\right)\times t$$

A plot of $\text{ln}\left(\frac{{\text{Target}}_{\text{rd}}{|}_{t}-{\text{Target}}_{\text{rd}}{|}_{ss}}{{\text{Target}}_{\text{rd}}{|}_{0}-{\text{Target}}_{\text{rd}}{|}_{ss}}\right)$ vs. time gives a linear relationship with slope = −(*k_switchoff_ + k_activation_*).

Finally, combining this slope with Equation (6), kinetic parameters are estimated as:

(9a) $$k_{switchoff}=-\text{slope}\times {\text{Target}}_{\text{reduced}}{|}_{ss}$$

(9b) $$k_{switchoff}=-\text{slope}-k_{switchoff}$$

### Simplification of the master equation

Simplified forms of Equation (4) that apply to specific experimental conditions may constitute a useful alternative to estimate kinetic parameters.

#### Absence of H_2_O_2_

On the assumption that *k_activation_* = 0, i.e., H_2_O_2_ is absent in the system, Equation (4) was simplified as Equation (10).

(10a) $${{\text{Target}}_{\text{rd}}|}_{t}-1=\left({\text{Target}}_{\text{rd}}{|}_{0}-1\right)\times e^{-k_{switchoff}\times t}$$

Or

(10b) $${{\text{Target}}_{\text{ox}}|}_{t}={\text{Target}}_{\text{ox}}{|}_{0}\times e^{-k_{switchoff}\times t}$$

This equation is applied to determine *k_switchoff_* from time courses that follow the return of the sensor protein to its reduced form. Taking the logarithmic of both sides of Equation (10b):

(11) $$\text{ln}\left({\text{Target}}_{\text{ox}}{|}_{t}\right)=\text{ln}\left({\text{Target}}_{\text{ox}}{|}_{0}\right)-k_{switchoff}\times t$$

A plot of ln(Target_ox_|_*t*_) vs. time produces a straight line with *k_switchoff_* = −slope.

#### No target reduction

Equation (12), another simplified form of Equation (4), was obtained by ignoring target reduction, i.e., *k_switchoff_* = 0.

(12) $${\text{Target}}_{\text{rd}}{|}_{t}={\text{Target}}_{\text{rd}}{|}_{0}\times e^{-k_{activation}\times t}$$

This equation is used to estimate *k_activation_* from short time courses when target reduction is still negligible. Taking the logarithmic of both sides of Equation (12):

(13) $$\text{ln}\left({\text{Target}}_{\text{rd}}{|}_{t}\right)=\text{ln}\left({\text{Target}}_{\text{rd}}{|}_{0}\right)-k_{activation}\times t$$

A plot of ln(Target_rd_|_*t*_) vs. time produces a straight line with *k_activation_* = −slope.

### Concentration studies

In all previous equations, H_2_O_2_ is a hidden variable that influences *k_activation_* and kinetic parameters are estimated from experiments in which the time course of the oxidation state of the target protein is followed. If cells are exposed to various concentrations of H_2_O_2_, kinetic parameters may also be estimated by following the variation of the oxidation state of the target protein as a function of the H_2_O_2_ concentration at a given time point. To this end, *k_activation_* was replaced by *k_target + H2O2_* × [H_2_O_2_] in Equation (4), forming Equation (14):

(14) $$\begin{array}{l} {{\text{Target}}_{\text{rd}}|}_{t}=\frac{k_{switchoff}}{k_{switchoff}+k_{target\;+\;H_{2}O_{2}}\times \left[{\text{H}}_{2}{\text{O}}_{2}\right]}\\ \;+e^{-\left(k_{switch}+k_{target+H_{2}O_{2}}\times \left[{\text{H}}_{2}{\text{O}}_{2}\right]\right)\times t}({\text{Target}}_{\text{rd}}{|}_{0}\\ \;-\frac{k_{switchoff}}{k_{switchoff}+k_{target\;+\;H_{2}O_{2}}\times \left[{\text{H}}_{2}{\text{O}}_{2}\right]}) \end{array}$$

For a known fixed *t*, a non-linear two-parameter fitting of Target_rd_ |_t_ vs. [H_2_O_2_] allows to estimate *k_switchoff_* and *k_target + H2O2_*. As before, if one of the two parameters is already known a one-parameter non-linear fitting may be used to determine the other parameter.

Concerning Equation (8), after specifying the H_2_O_2_ concentration explicitly this equation became:

(15) $$\begin{array}{l} \text{ln}\left(\frac{{\text{Target}}_{\text{rd}}{|}_{t}-{\text{Target}}_{\text{rd}}{|}_{ss}}{{\text{Target}}_{\text{rd}}{|}_{0}-{\text{Target}}_{\text{rd}}{|}_{ss}}\right)=-k_{switchoff}\times t-k_{target\;+\;H_{2}O_{2}}\\ \;\times t\times \left[H_{2}O_{2}\right] \end{array}$$

A plot of $\text{ln}\left(\frac{{\text{Target}}_{\text{rd}}{|}_{t}-{\text{Target}}_{\text{rd}}{|}_{ss}}{{\text{Target}}_{\text{rd}}{|}_{0}-{\text{Target}}_{\text{rd}}{|}_{ss}}\right)$ vs. [H_2_O_2_] gives a linear relationship with slope = −*k_target + H_2_O_2__× t* and intercept = −*k_switchoff_ × t*. In order to use this equation, the fraction of reduced target reached at steady-state (Target_rd_ |_ss_) must be known previously. Therefore, time courses are needed for each H_2_O_2_ concentration in order to obtain this value, which lessens the applicability of this equation.

In absence of target reduction, the equivalent of Equation (13) was deduced as:

(16) $$\begin{array}{l} \text{ln}\left({\text{Target}}_{\text{rd}}{|}_{t}\right)=\text{ln}\left({\text{Target}}_{\text{rd}}{|}_{0}\right)\\ \;-k_{target\;+\;H_{2}O_{2}}\times t\times \left[{\text{H}}_{2}{\text{O}}_{2}\right] \end{array}$$

A plot of ln(Target_rd_|_*t*_) vs. [H_2_O_2_] produces a straight line with slope = −*k_target + H_2_ O_2__ × t*.

Importantly, the [H_2_O_2_] in these equations refers to the intracellular [H_2_O_2_] that reacts with the target. Therefore, in order to estimate *k_target + H2O2_*, this concentration must be known. Intracellular [H_2_O_2_] attained when cells are exposed to extracellular H_2_O_2_ can be estimated from the gradient between extracellular and intracellular H_2_O_2_. If this gradient is unknown, then these equations may be applied with the extracellular H_2_O_2_ concentrations, but the value of *k_target + H2O2_* obtained is referred to extracellular H_2_O_2_ concentrations, with the true value being higher. If instead *k_target + H2O2_* is known *a priori*, equations may be used to estimate the gradient between extracellular and intracellular H_2_O_2_.

### Validation of equations

To test the validity of the equations, the following mathematical kinetic model was set up. This model simulates ideal experiments in which cells are exposed to extracellular H_2_O_2_ or are stimulated to produce endogenous H_2_O_2_ in a receptor-mediated process. A key characteristic of these virtual experiments is that the kinetic parameters concerning H_2_O_2_ signaling are known a priori, corresponding to the kinetic parameters introduced in the model. Thus, by fitting the virtual time courses to the equations deduced previously, the validity of the equations can be tested objectively. If the equations are valid, kinetic parameters obtained in these fittings should be similar to those used in the kinetic model. In addition, by varying several parameters of the model, namely those concerning the experimental set up describing H_2_O_2_ exposure, experimental conditions in which the equations are valid may be defined.

The model is described by the following differential equations, which take into account two compartments, one referring to the extracellular space (*V_out_*) and the other to the cell volume (*V_in_*). Multicompartmentation was implemented as described previously (Alves et al., 2006).

$$\begin{array}{l} \frac{d{\left[{\text{H}}_{2}{\text{O}}_{2}\right]}_{\text{out}}}{dt}={\text{H}}_{2}{\text{O}}_{2}\_\text{production}\_\text{out}\\ \;+{\text{H}}_{2}{\text{O}}_{2}\_\text{export}\times V_{in}/V_{out}-{\text{H}}_{2}{\text{O}}_{2}\_\text{import}\\ \frac{d{\left[{\text{H}}_{2}{\text{O}}_{2}\right]}_{\text{in}}}{dt}={\text{H}}_{2}{\text{O}}_{2}\_\text{production}\_\text{in}+{\text{H}}_{2}{\text{O}}_{2}\_\text{import}\\ \;\times V_{out}/V_{in}-{\text{H}}_{2}{\text{O}}_{2}\_\text{export}-\text{v}\_\text{GPx}-\text{v}\_\text{Target}\\ \frac{d\left[{\text{target}}_{\text{rd}}\right]}{dt}=\text{v}\_\text{switch}\_\text{off}-\text{v}\_\text{Target} \end{array}$$

Reactions considered in the model (Table 1) were: extracellular production of H_2_O_2_ (H_2_O_2__production_out), which simulates, for example, production of H_2_O_2_ by glucose oxidase added to the incubation medium; intracellular production of H_2_O_2_ (H_2_O_2__production_in), which simulates the endogenous production triggered by a receptor-mediated process; permeation of H_2_O_2_ across the plasma membrane into (H_2_O_2__import) and out of the cell (H_2_O_2__export); consumption of H_2_O_2_ by an antioxidant enzyme (v_Gpx) and by a sensor protein target (v_Target); and finally, the switch-off mechanism of the target protein (v_switch_off). Table 2 shows the parameters used. Although kinetics and respective rate constants are based on published values, this model does not intend to model a particular cell or a specific signaling pathway. Reactivities of the target and the antioxidant enzyme toward H_2_O_2_ were based on that of PTP1B (Barrett et al., 1999) and glutathione peroxidase (GPx) (Flohe, 1979; Forstrom and Tappel, 1979), respectively. Levels of GPx, permeability constant for H_2_O_2_ across the plasma membrane, *V_out_* and *V_in_* were taken from Antunes and Cadenas (2000). *k_switchoff_* was obtained from the lower range of values estimated in this work, based on previously published experiments. The resulting differential equations were solved numerically with PLAS (Voit, 1991). In the kinetic model, concentrations of Target_rd_ and Target_ox_ were used. The respective fractions were calculated subsequently so that simulation data could be analyzed with the equations deduced here. The parameter *k_activation_* is not a rate constant in the numerical model, but it was calculated as *k_target + H2O2_* × [H_2_O_2in_]; when [H_2_O_2in_] was not constant, for example when a bolus addition of H_2_O_2_ or the endogenous non-constant production of H_2_O_2_ was simulated, an average [H_2_O_2in_] was used.

**Table 1:** **Reactions and respective rate laws included in the kinetic model**.

**Reaction**	**Name**	**Rate law**
→ H_2_O_2out_	H_2_O_2__production_out	v_ H_2_O_2out_
→ H_2_O_2in_	H_2_O_2__production_in	*k_H_2_O_2in_* or *k_H_2_O_2in_* × sine(time/1200 × 3.14)
H_2_O_2out_ → H_2_O_2in_	H_2_O_2__import	*Ps* × *A/V_out_* × [H_2_O_2out_]
H_2_O_2in_ → H_2_O_2out_	H_2_O_2__export	*Ps* × *A/V_in_* × [H_2_O_2in_]
$H_{2}O_{\text{2in}}\overset{GPx}{\to }H_{2}O$	v_GPx	*k_GPx_* × [GPx] × [H_2_O_2in_]
H_2_O_2in_ + Target_rd_ → Target_ox_ + H_2_O	v_Target	*k_Target+H2O2_* × [Target_rd_] × [H_2_O_2in_]
Target_ox_ → Target_rd_	v_switch_off	*k_switchoff_* × ([Target_tot_] − [Target_rd_])

**Table 2:** **Parameter values used in the kinetic model**.

**Parameter**	**Value**	**Parameter**	**Value**
*Ps*	2.0 μm s^−1^	k_GPx_	6 × 10^7^ M^−1^ s^−1^
*A*	627 μm^2^	GPx	2 × 10^−7^ M
*V_in_*	1472 μm^3^	k_Target +H2O2_	40 M^−1^ s^−1^
*V_out_*	679 × *V_in_*	k_switchoff_	1 × 10^−3^ s^−1^
v_H_2_O_2out_	(0–23.4) × 10^−7^ M s^−1^	Target_tot_	8.3 × 10^−9^ M
kH_2_O_2in_	(0–5) × 10^−3^ M s^−1^		

*All simulations used these parameters values; for cases where a range of values is indicated, the actual value used in the simulation is indicated in the respective figure legend*.

## Results

As a first step to test the equations derived here, redox signaling experiments were simulated to generate data that was introduced into the equations in order to determine kinetic parameters. The validity of the equations was checked by comparing the kinetic parameters obtained with those used in the simulations.

### Validation of equations with simulated experiments

The exposure of cell cultures to extracellular H_2_O_2_ initiates cellular responses that differ from those caused by the intracellular release of H_2_O_2_ triggered by receptor-mediated mechanisms (Forman, 2007), being the main difference the additional signal transduction pathways initiated in the first case. Nevertheless, the control of H_2_O_2_ delivery achieved by the extracellular exposure makes this approach more suitable for the purpose of estimating kinetic parameters. We simulated both approaches with the kinetic model described in Theory and Methods, starting with the extracellular exposure to H_2_O_2_.

#### Extracellular addition of H_2_O_2_

Cells may be exposed to extracellular H_2_O_2_ either by bolus additions or by incubation with steady-state concentrations of H_2_O_2_. In the bolus addition, a single dose of H_2_O_2_ is added to cells, constituting the most common method of exposing cell cultures to extracellular H_2_O_2_. It has the advantage of simplicity, but the results obtained are strongly dependent on the specific assay conditions (Marinho et al., 2013a). Among other factors, cell density and, for adherent-growing cells, the volume of incubation media dramatically affect the results.

In the steady-state methodology, exposure to H_2_O_2_ is calibrated so that cells are exposed for a known concentration of H_2_O_2_ that remains constant during the assay. Although more complex, this approach has much better experimental reproducibility with the actual H_2_O_2_ concentration in the assay being independent of experimental conditions. The implementation of this methodology is described in detailed in (Covas et al., 2013; Cyrne et al., 2013; Marinho et al., 2013a).

***Steady-state***. In the deduction of the kinetic equations, *k_activation_* (*k_target + H2O2_* × [H_2_O_2_]) was considered to be a constant parameter, i.e., H_2_O_2_ was assumed to be constant with time. So, as a positive control we started by analyzing the results simulated with a steady-state incubation, a case in which the equations tested should be valid.

**Figure 1:**
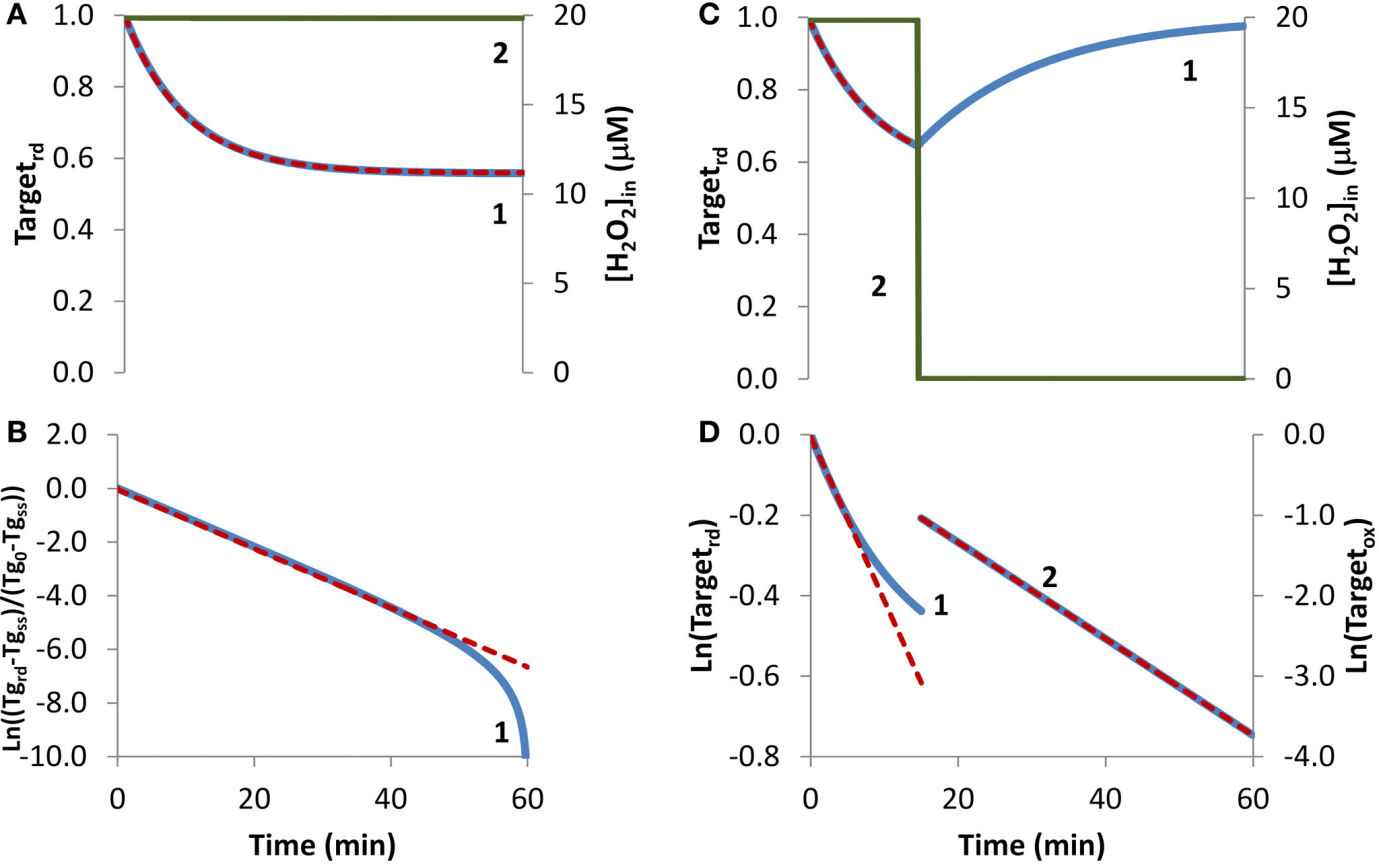
**Determination of kinetic parameters from simulated experiments when extracellular H_2_O_2_ was delivered as a steady-state**. H_2_O_2_ concentration (curve 2, green line) was maintained during the duration of the experiment **(A)** or was stopped at 15 min **(C)**. **(A)** The profile of the fraction of the H_2_O_2_ target in the reduced form (Target_rd_, blue line) was obtained by simulation of the kinetic model described in the Theory and Methods Section with v_H_2_O_2out_ = 3.51 × 10^−7^ M s^−1^ and [H_2_O_2out_] = 3 × 10^−4^ M at time = 0, (intracellular H_2_O_2_ production was absent, k_ H_2_O_2in_ = 0); red dashed line is the two-parameter non-linear fitting to Equation (4) used to estimate the kinetic parameters *k_activation_* and *k_switchoff_*. **(B)** Fitting of the profile of Target_rd_ obtained in **(A)** to Equation (8) (curve 1, blue line) using Target_rd_0_ = 1 and Target_rd_ss_ = 0.56, (Tg in y-axis title means Target). *k_activation_* and *k_switchoff_* were estimated from the slope of the straight line by applying Equations (9A) and (9B). **(C)** The profile of the fraction of Target_rd_ (curve 1, blue line) was obtained as in **(A)** but v_H_2_O_2out_ and [H_2_O_2_]_out_ were set to zero at 15 min; red dashed line is the one-parameter non-linear fitting to Equation (4) in which *k_switchoff_* obtained in **(D)** curve 2 was used as an input to estimate *k_activation_*. **(D)** Target_rd_ was fitted to Equation (13) (curve 1, left y-axis) while H_2_O_2_ was present, afterwards Target_ox_ was fitted to Equation (11) (curve 2, right y-axis); *k_activation_* was estimated from the linear part of the fitting to Equation (13), *k_switchoff_* from the fitting to Equation (11).

In the first condition analyzed, cellular exposure to H_2_O_2_ (Figure 1A, curve 2) was long enough so that a balance between oxidation of the target that senses H_2_O_2_ and its regeneration was achieved. As observed in Figure 1A, curve 1, initially the target was oxidized until its oxidation state reached a near steady-state value as given by Equation (6). Simulated results were transformed according to Equation (8) (Figure 1B, blue line), with *k_activation_* and *k_switchoff_* being estimated from Equations (9A) and (9B). The estimations obtained, *k_activation_* = 8.1 × 10^−4^ s^−1^ and *k_switchoff_* = 1.0 × 10^−3^ s^−1^, matched closely the respective expected values of 7.9 × 10^−4^ s^−1^ and 1.0 × 10^−3^ s^−1^, which were used in the simulations to draw curve 1 in Figure 1A. Note that the fitting to Equation (8) departed from linearity for longer time points (Figure 1B), when Target_rd_ approached its steady-state value (Figure 1B). This behavior was caused by small uncertainties in this value, which must be known in order to plot data according to Equation (8). As an alternative, the two parameters were also obtained from a non-linear fitting to Equation (4) (Figure 1A, red dashed line), which neither requires data transformation nor knowing the value of the fraction of reduced target at steady-state. In this case, the estimated parameters, *k_activation_* = 7.9 × 10^−4^ s^−1^ and *k_switchoff_* = 1.0 × 10^−3^ s^−1^, matched exactly the expected values.

If the fraction of the reduced target does not reach a steady-state because, for example, a balance between its oxidation and regeneration is not attained before the exposure to H_2_O_2_ is terminated, Equation (8) cannot be applied. To illustrate this situation, a simulation was done under the exact same conditions as before with the exception that H_2_O_2_ exposure lasted only for 15 min (Figure 1C). Experimentally, this is equivalent to either replacing the external media to remove the H_2_O_2_ generating system, such as glucose oxidase, or by adding external catalase to the incubation media. This simulation was analyzed with Equation (13), an equation deduced ignoring the regeneration of the reduced form of the target. Good enough estimations were obtained by using only the first stage of the time course, when the degree of target oxidation was still low, and therefore the contribution of its regeneration to the time course of Target_rd_, could be ignored. From the slope of the initial linear part of the curve (Figure 1D, curve 1), a *k_activation_* = 6.8 × 10^−4^ s^−1^ was obtained, which was close to the expected value of 7.9 × 10^−4^ s^−1^. Concerning *k_switchoff_*, this kinetic parameter was estimated by fitting to Equation (11) the part of the curve starting after removal of external H_2_O_2_. Note that Equation (11) was deduced assuming reduction of the oxidized target when H_2_O_2_ was absent. An excellent linear plot was observed (Figure 1D, curve 2) with the estimated *k_switchoff_* of 1.0 × 10^−3^ s^−1^ matching the expected value. Kinetic parameters were also obtained by non-linear fittings of Equation (4) to the first part of the curve when H_2_O_2_ was still present, either as a two-parameter non-linear fitting in which the two parameters – *k_activation_* and *k_switchoff_* – were determined, or as a one-parameter non-linear fitting, in which only one of the parameters was estimated, with the other being obtained from the linear plots of Figure 1B. If the conditions of the experimental assay fulfill all the assumptions applied to deduce Equation (4), a two-parameter non-linear fitting is the best choice, since both parameters are obtained without transformation of the experimental data. However, if the assumptions are not all fulfilled, which is the most common situation, we advise to apply a one-parameter non-linear fitting to Equation (4), inputting as a known parameter *k_switchoff_* estimated from Equation (11), being *k_activation_* the unknown parameter. As always whatever the option taken, the goodness of the fitting should be inspected. The dashed line in Figure 1C was obtained as a one-parameter non-linear fitting using *k_switchoff_* = 1.0 × 10^−3^ s^−1^ with the estimated *k_activation_* value of 7.9 × 10^−4^ s^−1^ matching the expected value.

Overall, kinetic parameters estimated from simulated experiments in which H_2_O_2_ was delivered as a steady-state matched the expected values, validating the equations applied. This could be anticipated because Equation (4) relies on the key assumption that H_2_O_2_ concentration is constant during the experiment.

**Figure 2:**
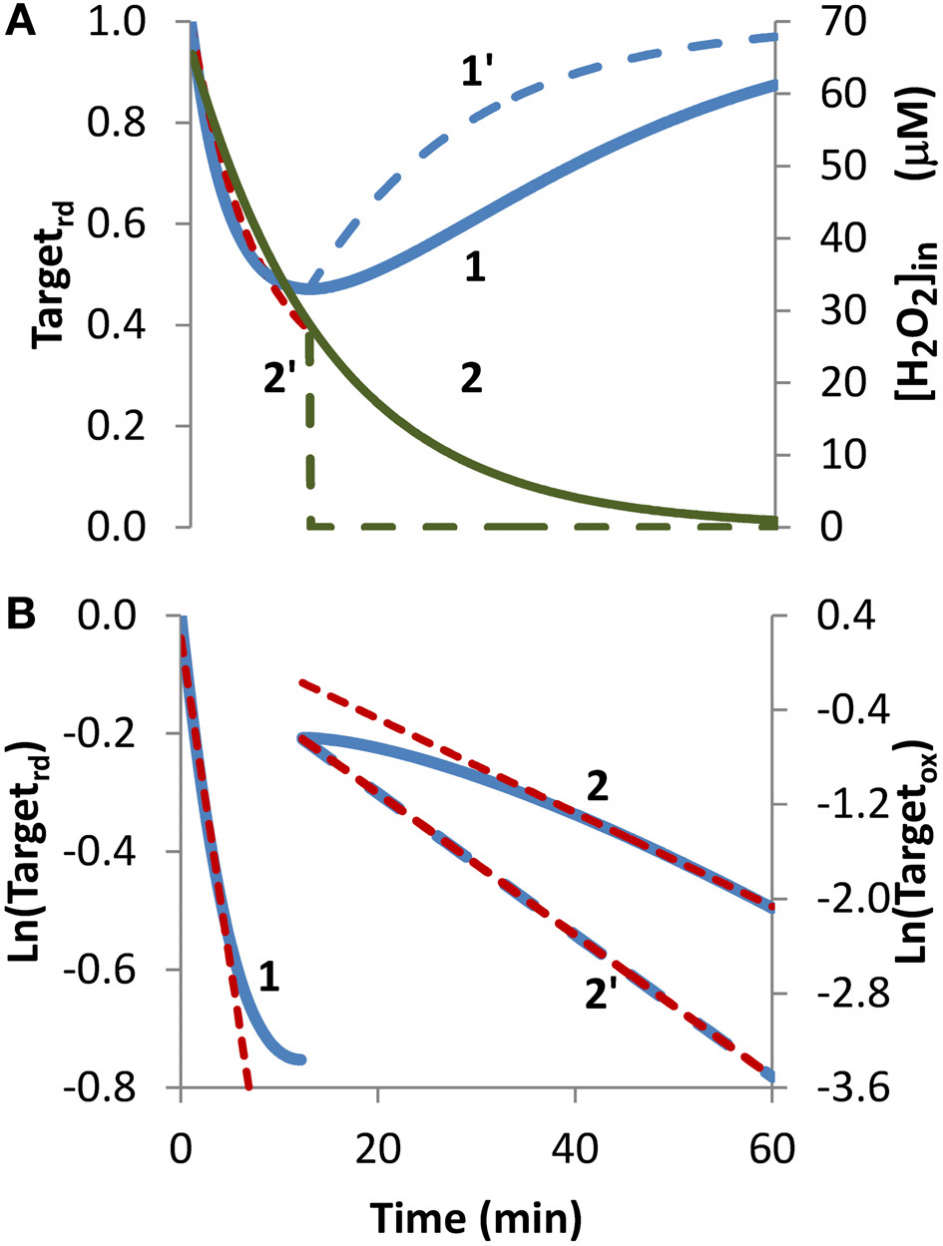
**Determination of kinetic parameters from simulated experiments when H_2_O_2_ was delivered as an extracellular bolus addition**. **(A)** Curve 1 is the profile of Target_rd_ fraction (blue line) obtained by simulation with v_H_2_O_2out_ = 0 M s^−1^ and [H_2_O_2out_] = 1 × 10^−3^ M at time = 0, (intracellular H_2_O_2_ production was absent, k_ H_2_O_2in_ = 0). Curve 1′ is the profile of Target_rd_ (blue line) obtained as in curve 1 but [H_2_O_2out_] was set to zero at 12 min; red dashed line is the one-parameter non-linear fitting to Equation (4) in which *k_switchoff_* obtained in **(B)** was used as an input to estimate *k_activation_*. **(B)** Until 12 min results were fitted to Equation (13) (curve 1, left y-axis), afterwards were fitted to Equation (11) (curve 2, right y-axis); *k_activation_* and *k_switchoff_* were estimated from the linear part of the fittings to Equations (13) and (11), respectively.

***Bolus addition***. The bolus addition set up, the most common experimental approach to expose cells to H_2_O_2_, was simulated in Figure 2. Upon incubation with a 1 mM bolus addition, the H_2_O_2_ sensor was oxidized, the Target_rd_ fraction reached a minimum at approximately 12 min, then regeneration became more important than oxidation, and Target_rd_ increased (Figure 2A, curve 1). Kinetics of H_2_O_2_ consumption depends on the experimental set up, and under the conditions of this simulation, H_2_O_2_ was fully consumed after 60 min (Figure 2A, curve 2). Nevertheless, the general pattern observed in this simulation served as a test case to check how the non-constant H_2_O_2_ concentration impacts the estimation of kinetic parameters.

Because the fraction of reduced target never reached a constant value, Equation (8) was not applied, and instead Equation (13) was used to estimate *k_activation_*. Only the first part of the curve was considered (Figure 2B, curve 1), because shorter time courses minimize target regeneration, a process ignored by Equation (13). The *k_activation_* estimation of 1.8 × 10^−3^ s^−1^ was close to the expected value of 1.7 × 10^−3^ s^−1^. Concerning *k_switchoff_*, this parameter was estimated by fitting to Equation (11) the part of the curve after Target_rd_ reached its minimum (Figure 2B, curve 2). The presence of H_2_O_2_ in this part of the experiment promoted target oxidation, violating a key assumption behind Equation (11), and consequently deviations from linearity were observed. Even by using only the linear part of the curve, the *k_switchoff_* estimation of 6.7 × 10^−4^ s^−1^ underestimated the expected value of 1.0 × 10^−3^ s^−1^. Removal of external H_2_O_2_ at 12 min, when Target_rd_ reached its minimum (Figure 2A, curve 2′), changed the regeneration profile of the H_2_O_2_ target (Figure 2A, curve 1′) and vastly improved the fitting to Equation (11) (Figure 2B, curve 2′) with the estimated *k_switchoff_* of 1.0 × 10^−3^ s^−1^ matching exactly the expected value.

Concerning the non-linear fitting to Equation (4), a *k_activation_* = 1.8 × 10^−3^ s^−1^ was obtained when *k_switchoff_* = 6.7 × 10^−4^ s^−1^ was used as an input. Alternatively, by inputting a *k_switchoff_* = 1.0 × 10^−3^ s^−1^ a *k_activation_* = 2.0 × 10^−3^ s^−1^ was obtained.

Overall, these results indicate that the proposed equations can be applied to experiments in which H_2_O_2_ is delivered as a bolus addition. The accuracy of parameter estimation improves if H_2_O_2_ is removed at the time when the reduced form of the target reaches its minimum.

**Figure 3:**
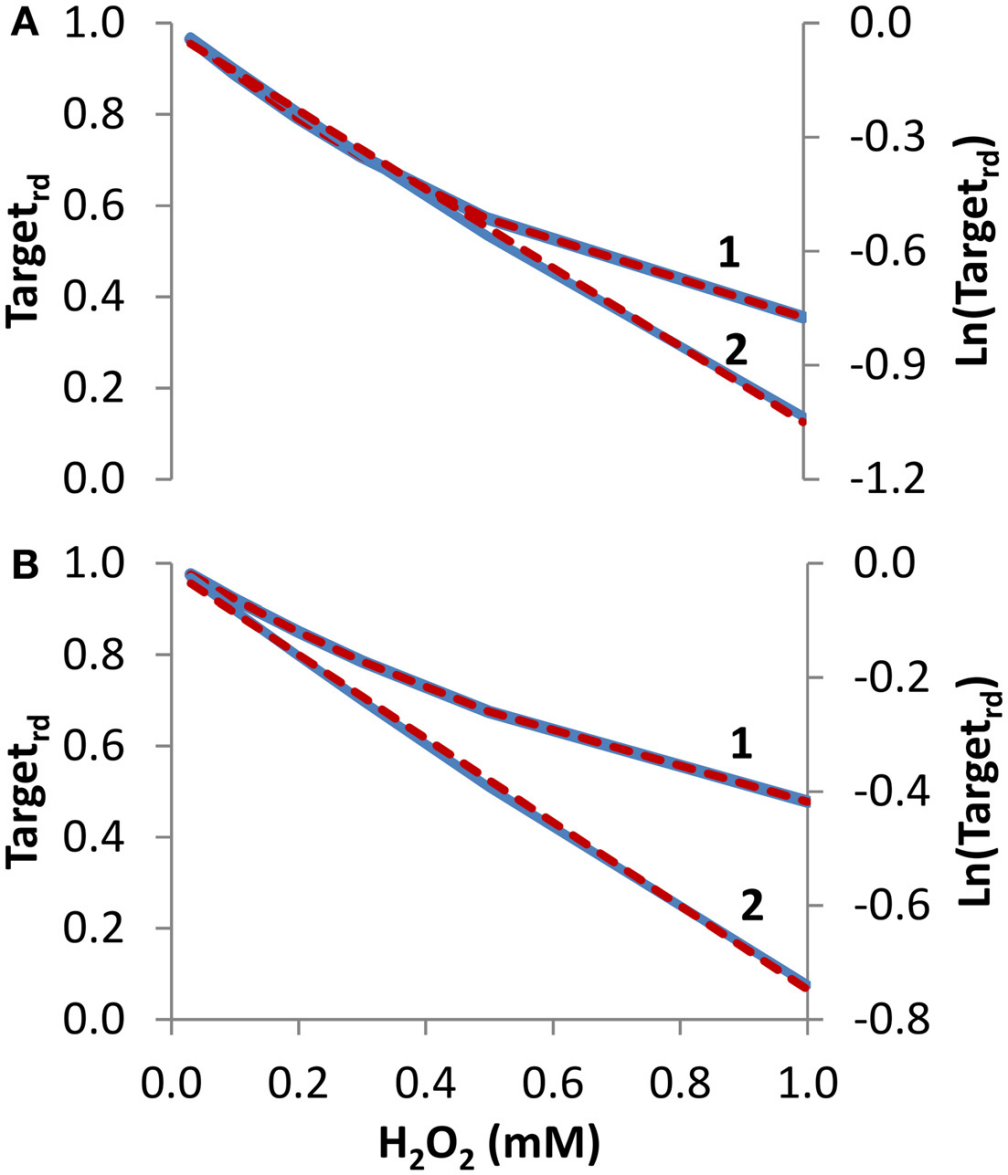
**Determination of kinetic parameters from simulated experiments when the concentration of extracellular H_2_O_2_ was changed**. The reduced form of the target fraction (Target_rd_) obtained at 10 min is plotted as a function of either H_2_O_2_ steady-state concentrations in **(A)** or initial bolus additions in **(B)** (curves 1, blue linen). Simulations were run with v_H_2_O_2out_ varying in the range (0.35–23.4) × 10^−7^ M s^−1^ and [H_2_O_2out_] in the range (0.03–1) × 10^−3^ M at time = 0 in A, while in B v_H_2_O_2out_ was set to zero and [H_2_O_2out_] was changed in the range (0.03–1) × 10^−3^ M at time = 0; intracellular H_2_O_2_ production was absent (k_ H_2_O_2in_ = 0) in both cases. In **(A,B)**, results were analyzed with non-linear fits of Target_rd_ to Equation (14) (curve 1, red dashed line) in order to estimate *k_target + H2O2_* and *k_switchoff_*, or they were linearized according to Equation (16) (curve 2, blue line) with *k_target + H2O2_* being estimated from the slopes of the red dashed lines.

***Concentration studies***. Besides time courses, studies often evaluate how the concentration of H_2_O_2_ affects the oxidation state of the sensor target. We started by simulating the dependency of Target_rd_ on external H_2_O_2_ concentration, delivered as a steady-state during 10 min (Figure 3A, curve 1). From the non-linear fitting to Equation (14), kinetic parameters that matched exactly the expected values were obtained (*k_target + H2O2_* = 2.6 M^−1^ s^−1^ and *k_switchoff_* = 1.0 × 10^−3^ s^−1^). Note that *k_target + H2O2_* estimated from the fitting was based on external H_2_O_2_ concentrations. By considering the gradient between these and the intracellular H_2_O_2_ concentrations—15 in the present simulation—the estimated value of the rate constant of 40 M^−1^ s^−1^ for the reaction between the target and H_2_O_2_ matched the value used in the simulation. Results were also linearized and fitted to Equation (16) (curve 2 in Figure 3A), which was deduced assuming absence of target reduction, i.e., *k_switchoff_* = 0 s^−1^. In this case, the estimated value of 1.7 M^−1^ s^−1^ for *k_target + H2O2_*, which was converted to 26 M^−1^ s^−1^ when intracellular H_2_O_2_ concentrations were considered, underestimated the expected value.

To test how Equations (14) and (16) behave with data generated with bolus additions, the study of Figure 3A was repeated but now the H_2_O_2_ concentrations introduced in the equations were the initial bolus additions (Figure 3B, curve 1). Kinetic parameters obtained with the non-linear fitting to Equation (14) were *k_target + H2O2_* = 2.0 M^−1^ s^−1^ (or 30 M^−1^ s^−1^ if referred to intracellular H_2_O_2_), and *k_switchoff_* = 1.3 × 10^−3^ s^−1^. Linearization according to Equation (16) (curve 2 in Figure 3B) gave a *k_target + H2O2_* of 1.2 M^−1^ s^−1^, (or 18 M^−1^ s^−1^ if referred to intracellular H_2_O_2_). As expected, these estimations were less accurate than those obtained when H_2_O_2_ was delivered as a steady-state, but nevertheless they constitute satisfactory semi-quantitative estimations.

#### Receptor-mediated endogenous H_2_O_2_ production

The endogenous production of H_2_O_2_ upon cell stimulation by a ligand will give the best picture of the influence of H_2_O_2_ in a particular cell signaling pathway, as H_2_O_2_ production is both spatial and time restricted (Forman, 2007). Nevertheless, since the profile of H_2_O_2_ concentration generated is unknown this imposes potential problems to the determination of kinetic parameters. To test how the kinetic equations behave under such circumstances, we started by simulating a case where H_2_O_2_ intracellular production was rapidly triggered and then set at a near constant value. This scenario worked as positive control and was analyzed as described previously for the extracellular addition of steady-state H_2_O_2_. The kinetic parameters obtained matched exactly the expected values or were very close to these values depending on the fittings applied (not shown). Nevertheless, this scenario is seldom achieved when H_2_O_2_ is produced endogenously, and next we tested the kinetic equations under non-constant H_2_O_2_ intracellular production.

**Figure 4:**
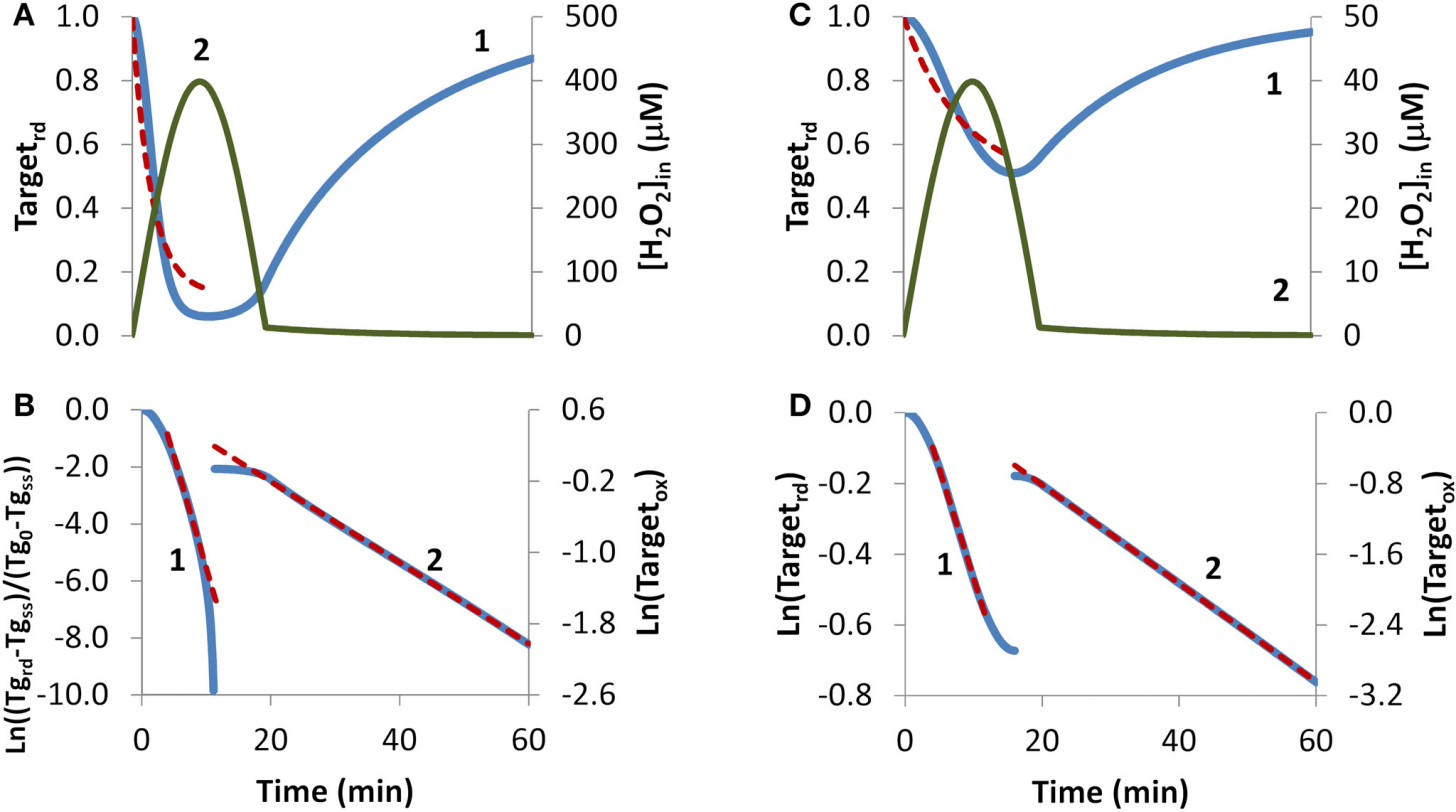
**Determination of kinetic parameters from simulated experiments when H_2_O_2_ was produced endogenously**. A non-constant sine-like H_2_O_2_ intracellular exposure (green lines) was simulated as kH_2_O_2in_ × sine(time/1200 × 3.14), (extracellular production was absent, v_H_2_O_2out_ = 0). **(A)** Profile of Target_rd_ fraction (blue line) was simulated with kH_2_O_2in_ = 5 × 10^−3^ M s^−1^; red dashed line is the one-parameter non-linear fitting to Equation (4) in which *k_switchoff_* obtained in **(B)** curve 2 was used as an input to estimate *k_activation_*. **(B)** The first part of the profile (until 11.3 min) of Target_rd_ was fitted to Equation (8) (curve 1, blue line) using Target_rd_0_ = 1 and Target_rd_ss_ = 0.06; the kinetic parameters *k_activation_* and *k_switchoff_* were obtained from the slope of near-linear intermediate portion of the curve by applying Equations (9A) and (9B). After 11.3 min, the profile of Target_ox_ was fitted to Equation 13, from which *k_switchoff_* was estimated. **(C)** The profile of Target_rd_ fraction (blue line) was obtained as in **(A)**, but with kH_2_O_2in_ = 0.5 × 10^−3^ M s^−1^; red dashed line is the one-parameter non-linear fitting to Equation (4) in which *k_switchoff_* obtained in **(D)** curve 2 was used as an input to estimate *k_activation_*. **(D)** Target_rd_ was fitted to Equation (13) (curve 1, left y-axis) until 16.0 min, afterwards, Target_ox_ was fitted to Equation (11) (curve 2, right y-axis); *k_activation_* and *k_switchoff_* were estimated from the linear part of the fittings to Equations (13) and (11), respectively.

H_2_O_2_ endogenous production was simulated with a sine-like function: there was an initial increase in the H_2_O_2_ concentration, reaching its maximum at 10 min, and then a decrease until H_2_O_2_ production stopped at 20 min (Figures 4A,C, curve 2). In this context, two scenarios were simulated. In the first, H_2_O_2_ production was high enough so that a near constant level of reduced target was observed (Figure 4A, curve 1), and accordingly results were fitted to Equation (8). The presence of a non-constant H_2_O_2_ production caused deviations from linearity (Figure 4B, curve 1). Nevertheless results obtained from the near-linear intermediate portion of the curve gave estimations, *k_switchoff_* = 7.8 × 10^−4^ s^−1^ and *k_activation_* = 1.2 × 10^−2^ s^−1^, that compared well with the expected parameters, *k_switchoff_* = 1.0 × 10^−3^ s^−1^ and *k_activation_* = 1.0 × 10^−2^ s^−1^. As before, *k_switchoff_* was also obtained from the second part of the curve by fitting data to Equation (11) (Figure 4B, curve 2), giving a *k_switchoff_* = 7.6 × 10^−4^ s^−1^, an underestimation of the expected value. As described for the bolus addition, removal of H_2_O_2_ from the system after Target_rd_ reached its minimum improved the estimations (not shown). In real experiments, the effect of this addition will be dependent on whether removal of extracellular H_2_O_2_ decreases the localized intracellular levels of H_2_O_2_. If this occurs, a change in the reduction profile of the oxidized target should be observed. In this simulation, the application of non-linear fittings did not improve the estimations of kinetic parameters: from a non-linear fitting where *k_switchoff_* = 7.6 × 10^−4^ s^−1^ was used as input (dashed line in Figure 4A) a *k_activation_* of 0.51 × 10^−2^ s^−1^ was obtained (Figure 4A, dashed line) and a two-parameter non-linear fitting did not improve these estimations.

In the second simulation in which endogenous production of H_2_O_2_ followed a sine-like function, a constant level of reduced target was not observed (Figure 4C, curve 1). The *k_activation_* estimation of 1.0 × 10^−3^ s^−1^, obtained from the linear portion of the plot according to Equation (13) (Figure 4D, curve 1), matched the expected value. The *k_switchoff_* estimation of 9.3 × 10^−4^ s^−1^, obtained from the second part of the curve after fitting data to Equation (11) (Figure 4D, curve 2), was close to the expected value of 1.0 × 10^−3^ s^−1^. A one-parameter non-linear fitting (Figure 4C, dashed line) gave a *k_activation_* of 1.0 × 10^−3^ s^−1^, i.e., the expected value, when a *k_switchoff_* of 9.3 × 10^−4^ was used as input.

Overall, when H_2_O_2_ production is not constant deviations from the equations derived here are expected. Nevertheless, estimated kinetic parameters are still satisfactory at a semi-quantitative level, and the deviations from linearity in the plots proposed here may be used as a useful tool to diagnose a non-constant H_2_O_2_ production.

### Fits to experimental data

The use of simulation data was useful to test the validity of the equations deduced and to figure out how deviations from the assumptions behind their deduction affected the estimation of kinetic parameters. Nevertheless, simulation data points are virtually infinite and devoid of experimental error; in contrast real experiments contain a finite number of measurements with associated experimental error. To test how equations cope with these issues, they were applied to data obtained from the literature for two PTPs, PTP1B and SHP-2.

#### H_2_o_2_-external delivery

**Figure 5:**
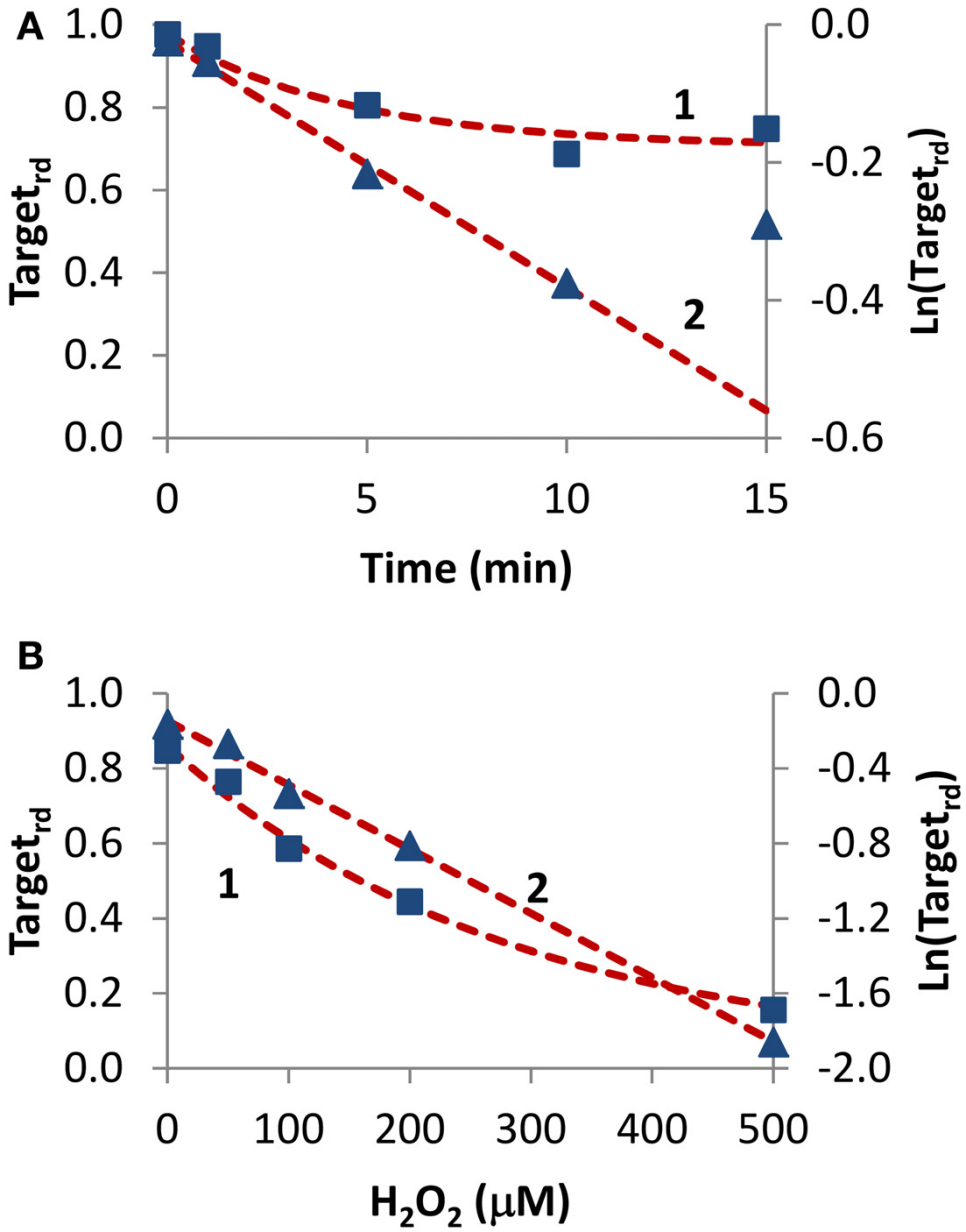
**Determination of kinetic parameters from experiments when H_2_O_2_ was delivered as an extracellular bolus addition**. **(A)** Data (■) was taken from Figure 1 in Rinna et al. (2006). Gel was digitalized and analyzed with ImageJ (Rasband, 1997); fraction of oxidized target was calculated assuming a maximum level of oxidation of 31% (Rinna et al., 2006). Time course was fitted to Equation (4), with *k_activation_* and *k_switchoff_* as variables (curve 1, left y-axis), or was linearized (▲) and fitted to Equation (13) (curve 2, right y-axis). **(B)** Data (■) was taken from Figure 2A in Meng et al. (2002). Gel was digitalized and analyzed with ImageJ (Rasband, 1997); fraction of oxidized protein tyrosine phosphatase corresponding to 70 kDa (SHP-2) was calculated assuming that complete oxidation was achieved by 1 mM H_2_O_2_. Time course was fitted to Equation (14), with *k_target + H2O2_* and *k_switchoff_* as variables (curve 1, left y-axis), or was linearized (▲) and fitted to Equation (16) (curve 2, right y-axis).

Two experiments in which H_2_O_2_ was added externally as a bolus addition (Figure 5) were analyzed. In the first experiment (Rinna et al., 2006), the time course of PTP1B oxidation was followed in a rat alveolar macrophage cell line after addition of a 100 μM H_2_O_2_ bolus dose for 15 min (Figure 5A, curve 1). By fitting data to Equation (4) with a two parameter non-linear fitting, a *k_activation_* of 1.1 × 10^−3^ s^−1^ and a *k_switchoff_* of 2.6 × 10^−3^ s^−1^ were estimated. Alternatively, when results were linearized according to Equation (13) (Figure 5A, curve 2) a *k_activation_* of 0.59 × 10^−3^ s^−1^ was obtained, after discarding the 15 min point. Note that also with simulation data a similar deviation from linearity at late time points was observed (Figure 2B, curve 1). By considering an external H_2_O_2_ concentration of 100 μM, the apparent first-order rate constant *k_activation_* in the range (0.59–1.1) × 10^−3^ s^−1^ was converted to a rate constant between the target and H_2_O_2_ (*k_target + H2O2_*) of 5.9–11 M^−1^ s^−1^. This value refers to extracellular H_2_O_2_, and so if the gradient between extracellular and intracellular H_2_O_2_ was considered the value of *k_target + H2O2_* for PTP1B would be higher.

In the second experiment, rat-1 fibroblasts were subjected to H_2_O_2_ bolus additions in the range 0–500 μM for 1 min, followed by the measurement of the oxidation level of SHP-2 (Meng et al., 2002) (Figure 5B, curve 1). After fitting data to Equation (14) (Figure 5B, curve 1) with a two parameter non-linear fitting, a *k_target + H2O2_* of 60 M^−1^ s^−1^ and a *k_switchoff_* of 1.3 × 10^−3^ s^−1^ were estimated. Linearization according to Equation (16) gave a *k_target + H2O2_* of 57 M^−1^ s^−1^ (Figure 5B, curve 2). Again, *k_target + H2O2_* values refer to extracellular H_2_O_2_ concentrations. Even if a bolus addition was used, because short-term incubations of 1 min were done, the assumption of constant H_2_O_2_ behind the deduction of Equations (14) and (16) was verified.

#### Receptor-mediated signaling

**Figure 6:**
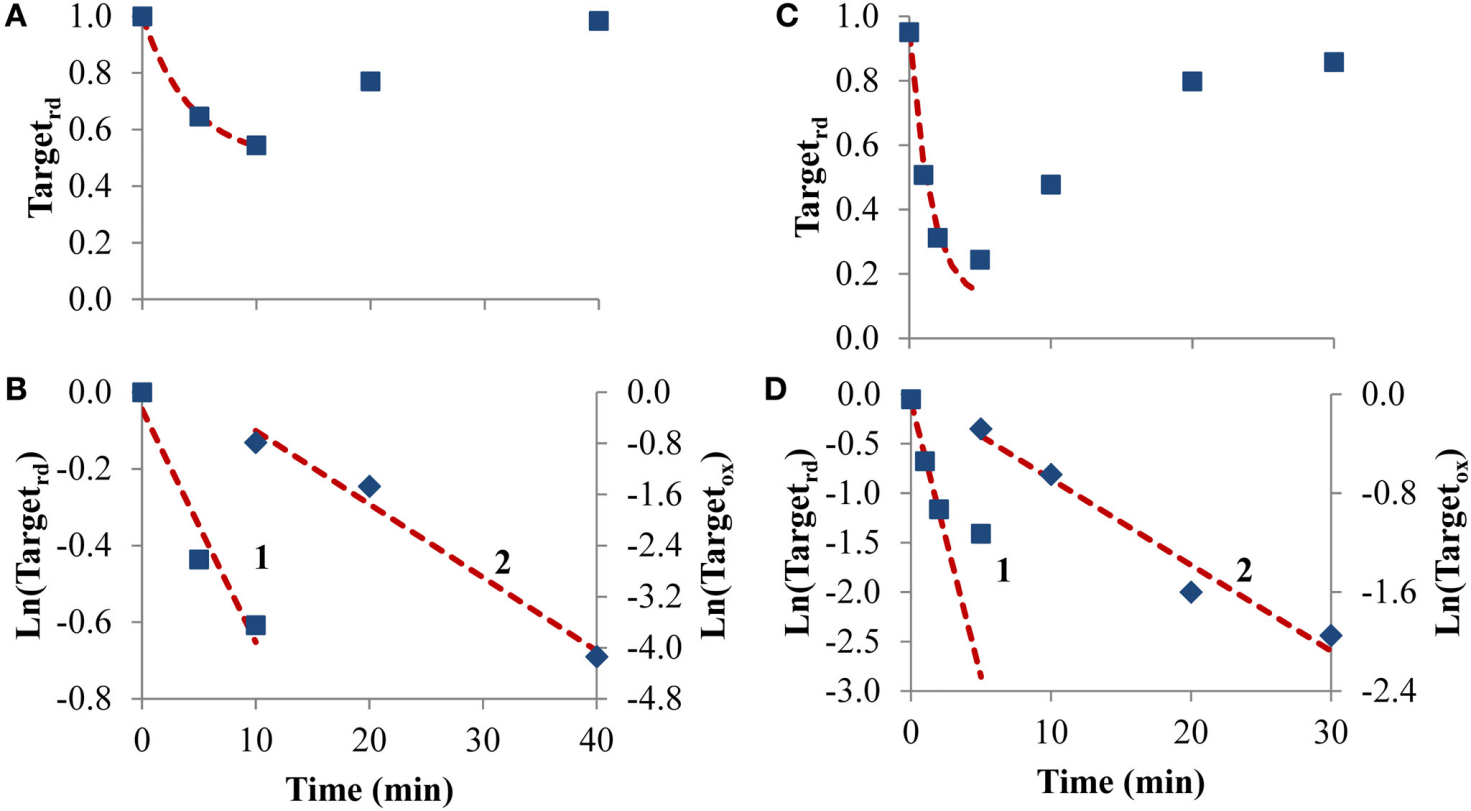
**Determination of kinetic parameters from experiments where intracellular H_2_O_2_ production was triggered by cell stimulation**. **(A)** Data concerning PTP1B oxidation following stimulation of A431 cells with EGF (■) was taken from Figure 2 in Lee et al. (1998). **(C)** Data concerning SHP-2 oxidation following stimulation of rat-1 cells by PDGF (■) was taken from gel in Figure 4 in Meng et al. (2002) after digitalization and analysis with ImageJ (Rasband, 1997), and assuming that 100% of oxidation of SHP-2 was achieved in absence of iodoacetic acid as described in Meng et al. (2002). The red dashed lines in **(A,C)** are one-parameter non-linear fittings to Equation (4) in which *k_switchoff_* values obtained from curves 2 in **(B,D)**, respectively, were used as input to estimate *k_activation_*. **(B,D)** Linearization of data shown in **(A,C)**, respectively; results in the first part of the experiment were fitted to Equation (13) (curves 1, left y-axis), afterwards were fitted to Equation (11) (curves 2, right y-axis); *k_activation_* was estimated from the fitting to Equation (13), *k_switchoff_* from the fitting to Equation (11).

To test how equations behave when analyzing receptor-mediated signaling, the following two experiments were considered. In the first, A431 human epidermoid carcinoma cells were stimulated by EGF, triggering H_2_O_2_ intracellular production that lead to PTP1B oxidation and inhibition (Figures 6A,B), while in the second experiment, rat-1 cells were stimulated with PDGF inducing SHP-2 oxidation (Figures 6C,D). In both cases, the profile of PTP oxidation did not reach a near steady-state, precluding the application of Equation (8). Concerning *k_switchoff_*, estimations of 1.9 × 10^−3^ s^−1^ and 8.7 × 10^−3^ s^−1^ were obtained, respectively for PTP1B and SHP-2 reactivation, after fitting to Equation (11) the second part of the PTP oxidation curves (curves 2 in Figures 6B,D). For *k_activation_*, estimations of 1.0 × 10^−3^ s^−1^ and 9.3 × 10^−3^ s^−1^ were obtained, respectively for PTP1B and SHP-2, after applying Equation (13) to linearize the first part of the PTP oxidation profile (curve 1 in Figures 6B,D). These *k_activation_* values were close to those obtained from non-linear fittings, 2.0 × 10^−3^ s^−1^ and 9.7 × 10^−3^ s^−1^ for PTP1B and SHP-2, respectively (dashed lines in Figures 6A,C).

Overall, data taken from literature fitted well to the equations deduced here, even if experiments analyzed were carried out without any special concern considering their application to estimate kinetic parameters.

## Discussion

Herein, we deduced equations to determine kinetic parameters from typical redox signaling experiments in which H_2_O_2_ is either added externally to cells or is endogenously produced following receptor activation by diverse cellular stimuli. The equations were shown to be accurate after fitting them to data generated by simulations. We also performed simulations in which the assumption that H_2_O_2_ is constant during the experiment was not fulfilled, that is, H_2_O_2_ was delivered as a bolus addition, or the endogenous production of H_2_O_2_ was not constant. Under these conditions, deviations from linearity were observed when simulation results were plotted according to the linear equations we deduced. Nevertheless, the estimated kinetic parameters were close to the parameters introduced in the simulations. Finally, we tested the application of the equations to real experiments with published experimental data concerning the H_2_O_2_ signaling mediated by inhibition of PTPs, namely PTP1B and SHP-2. While in general excellent fittings were obtained, in some cases deviations as those observed when H_2_O_2_ was added as a bolus addition were observed. In general, the estimated kinetic parameters (Table 3) are consistent with the published rate constants.

**Table 3:** **Kinetic parameters estimated in this work based on the analysis of published data**.

***PTP***	***k_switchoff_***		***k_target_ + H2O2***	***k_activation_***
	**External H_2_O_2_**	**Receptor-mediated H_2_O_2_ production**	**External H_2_O_2_**	**Receptor-mediated H_2_O_2_ production**
*PTP1B*	2.6 × 10^−3^ s^−1^	1.9 × 10^−3^ s^−1^	5.9–11 M^−1^ s^−1^	(1–2) × 10^−3^ s^−1^
*SHP-2*	1.3 × 10^−3^ s^−1^	8.7 × 10^−3^ s^−1^	57–60 M^−1^ s^−1^	(9.3–9.7) × 10^−3^ s^−1^

*Apparent first-order rate constant for the reactivation of PTP (k_switchoff_), the rate constant for the inactivation of PTP by extracellular H_2_O_2_ (k_target +H2O2_), and the apparent first-order rate constant for this activation (k_activation_ = k_target+H2O2_ × [H_2_O_2_]) are shown*.

Concerning the parameters describing redox signal switching-off (*k_switchoff_*), which in the case of the PTPs analyzed here corresponds to their reactivation, the results summarized in Table 3 constitute, as far as we know, the first cell-based kinetic estimates for this process. This characterization is relevant because modulation of PTP reactivation regulates cell signaling (Dagnell et al., 2013). For PTP1B, *k_switchoff_* values in the range (1.9–2.6) × 10^−3^ s^−1^ were estimated, which are plausible taking into account the known data for PTP1B reactivation. *In vitro*, reduced thioredoxin (2 μM) reactivates oxidized PTP1B with an apparent rate constant of 1.4 × 10^−3^ s^−1^ (Parsons and Gates, 2013), which corresponds to a rate constant of 700 M^−1^ s^−1^ for this reaction. Thus, considering this rate constant and the *k_switchoff_* values determined here, we estimate the cellular concentration of reduced thioredoxin to be 2–3 μM. This range is close to the value observed experimentally in Jurkat T-cells, 0.43 μM (Adimora et al., 2010), with the difference observed being easily account for different cell lines used or by the participation of alternative thioredoxin-related proteins, like the redoxin TRP14, in the reactivation of PTP1B (Dagnell et al., 2013). This agreement further strengths the validity of the approach we purpose here to reveal kinetic information hidden in typical redox signaling experiments.

For SHP-2, *k_switchoff_* was estimated in the range (1.3–8.7) × 10^−3^ s^−1^ (Table 3), which is similar or higher than the range estimated for PTP1B. This is unexpected because the reactivity of SHP-2 toward thioredoxin is about 20 times lower than PTP1B (Parsons and Gates, 2013). Either the cell line rat-1, where SHP-2 reactivation data was obtained (Meng et al., 2002), contains much higher levels of thioredoxin or an alternative system other than thioredoxin is reactivating SHP-2. The second alternative is supported by the observation that in cells lacking thioredoxin reductase TrxR1, a key partner of thioredoxin that keeps it in the reduced state, SHP-2 oxidation remains unchanged (Dagnell et al., 2013).

Concerning the oxidation of PTPs by H_2_O_2_, rate constants estimated from experiments in which extracellular H_2_O_2_ was added were 5.9–11 M^−1^ s^−1^ for PTP1B and 57–60 M^−1^ s^−1^ for SHP-2 (Table 3). These values were calculated based on the external H_2_O_2_ concentrations added to cells. The actual H_2_O_2_ concentration that oxidizes these targets is lower as H_2_O_2_ gradients across the plasma membrane are established when cells are incubated with extracellular H_2_O_2_ (Antunes and Cadenas, 2000; Marinho et al., 2013b). Thus, the value of these rate constants will be higher if they are based on the actual intracellular H_2_O_2_ concentrations that oxidize PTPs. For PTP1B, rate constants obtained in kinetic studies with purified PTP1B are in the range 9–43 M^−1^ s^−1^ (Denu and Tanner, 1998; Barrett et al., 1999; Zhou et al., 2011; Marinho et al., 2014), and so a gradient between the extracellular and the intracellular concentration of H_2_O_2_ at the site of PTP1B oxidation is estimated to be in the range 2–7 for the experiments analyzed in this work, which matches the lower range of known gradients for human cell lines (Antunes and Cadenas, 2000; Makino et al., 2004; De Oliveira-Marques et al., 2007; Oliveira-Marques et al., 2013). However, gradients as high as 650 have been recently estimated taking into account the participation of peroxiredoxin (Huang and Sikes, 2014), whose role in the degradation of H_2_O_2_ is still an open issue (Benfeitas et al., 2014). Thus, the rate constants obtained for PTP1B fit the known quantitative data for the reactivity of this PTP with H_2_O_2_.

For SHP-2, the estimated rate constants of 57–60 M^−1^ s^−1^ (Table 3) for its oxidation by H_2_O_2_ were higher than those determined *in vitro* with purified SHP-2, which are in the range 9–15 M^−1^ s^−1^ (Chen et al., 2009; Zhou et al., 2011). Moreover, if the gradient of H_2_O_2_ across the plasma membrane is taken into account this difference will be even higher. Several possible explanations may account for this discrepancy. First, kinetic rate constants obtained *in vitro* with purified proteins may not reflect rate constants under *in vivo* conditions (Van Eunen et al., 2010, 2012). Second, peroxy-derivatives such as peroxymonocarbonate (Trindade et al., 2006; Zhou et al., 2011) and peroxymonophosphate (LaButti et al., 2007), which have higher reactivity with PTPs than H_2_O_2_, could be the actual species that oxidize SHP-2. Third, the primary sensor of H_2_O_2_ may not be SHP-2 but a high-reactive target that slowly relays the oxidation to SHP-2 (Winterbourn and Hampton, 2008; Forman et al., 2010; Brigelius-Flohé and Flohé, 2011; Ferrer-Sueta et al., 2011). Note that the models described here do not distinguish between a mechanism in which a low-reactive sensor is slowly oxidized by H_2_O_2_, from a mechanism in which a high-reactive sensor is rapidly oxidized by H_2_O_2_ and then, through a thiol-disulfide reshuffling transfer reaction, slowly oxidizes a low reactive sensor such as SHP-2. In general, known data about redox signaling pathways is consistent with either of these two scenarios (Marinho et al., 2014). Distinguishing between these possible alternative mechanisms will be possible after collecting rate constants in several cell lines upon the generalized application of the equations deduced here to redox signaling experiments.

The kinetic parameters estimated from experiments in which cells are activated by receptor-mediated pathways indicated that the apparent first-order rate constant for the oxidation of SHP-2 is about 5 times higher than that for PTP1B (Table 3). Because *k_activation_* = k_target + H2O2_ × [H_2_O_2_], either the localized H_2_O_2_ intracellular concentration is higher in the experiment in which SHP-2 oxidation was observed, or *k_target + H2O2_* is higher for SHP-2 than for PTP1B, or both. In this regard, the EGF receptor, the H_2_O_2_ producing enzyme NOX2, and SHP-2 immunoprecipitated all together (Paulsen et al., 2012), supporting the possibility of a highly localized H_2_O_2_ signaling pool. For PTP1B, from the *k_activation_* estimation of (1.0–2.0) × 10^−3^ s^−1^ the local intracellular H_2_O_2_ concentration reached locally in A431 cells, when stimulated by EGF under the experimental conditions described in Lee et al. (1998), is estimated to be in the range 23–220 μM, assuming a *k_target + H2O2_* value in the range 9–43 M^−1^ s^−1^. Such local concentrations, particularly those in the low range of these values, can potentially be reached upon the concerted action of local production of H_2_O_2_ by NADPH oxidases (Chen et al., 2008; Mishina et al., 2011; Paulsen et al., 2012) and localized inhibition of H_2_O_2_ removing enzymes (Woo et al., 2010; Rawat et al., 2013). In addition, it can also be suggested that H_2_O_2_ diffusion out of membrane-entrapped signaling microcompartments may be constrained, because biomembranes constitute a regulable barrier for H_2_O_2_ diffusion (Antunes and Cadenas, 2000; Branco et al., 2004; Bienert et al., 2007; Miller et al., 2010).

While the equations deduced here were applied successfully to typical signaling experiments, a few alterations in the way experiments are carried out will improve the accuracy of parameter estimation. When cells are exposed to extracellular H_2_O_2_, we suggest a steady-state delivery so that H_2_O_2_ is constant during the experiment (Marinho et al., 2013a), a key assumption considered in the deduction of the equations. If the use of a bolus addition is absolutely needed, we suggest short-term experiments so that the H_2_O_2_ decay caused by its cellular consumption is less significant. Finally, removal of H_2_O_2_ by adding catalase or replacing extracellular incubation media without H_2_O_2_, during the second part of the experiment when target reduction starts to predominate, improves the estimation of *k_switchoff_* values. This last suggestion may also be applied when H_2_O_2_ production is triggered by a receptor-mediated mechanism following cell stimulation.

In conclusion, the application of the equations deduced here to typical redox-signaling experiments reveals valuable quantitate kinetic information. Of note, the equations described require only measuring the relative levels of oxidation of a H_2_O_2_ sensor target and not absolute concentrations, thus facilitating their application to most experiments. While equations were tested with PTP signaling, they can be applied to other proteins that react with H_2_O_2_, such has thiol-proteins and those containing metal-centers. Being characterized by the presence of both multiple parallel pathways and biphasic effects, redox regulation is a field that will benefit from the widespread determination of kinetic parameters. Such knowledge is important to distinguish apparent contradictory biological effects of reactive oxygen species that are involved in pathological damaging pathways and, at the same time, are part of normal functional signaling pathways. In this way, the present knowledge on redox signaling and oxidative stress would be more efficiently translated into therapeutic applications.

### Conflict of interest statement

The authors declare that the research was conducted in the absence of any commercial or financial relationships that could be construed as a potential conflict of interest.

## Abbreviations

PTP1B: Protein tyrosine phosphatase 1
PTPs: protein tryrosine phosphatases; the Src homology 2 (SH2) domain containing phosphotyrosine phosphatase 2 (Shp-2).

## References

[B1] Adimora N. J. Jones D. P. Kemp M. L. (2010). A model of redox kinetics implicates the thiol proteome in cellular hydrogen peroxide responses. Antioxid. Redox Signal. 13, 731–743 10.1089/ars.2009.2968 20121341 PMC2935341

[B2] Alves R. Antunes F. Salvador A. (2006). Tools for kinetic modeling of biochemical networks. Nat. Biotechnol. 24, 667–672 10.1038/nbt0606-667 16763599

[B3] Antunes F. Cadenas E. (2000). Estimation of H2O2 gradients across biomembranes. FEBS Lett. 475, 121–126 10.1016/S0014-5793(00)01638-0 10858501

[B4] Barrett W. C. DeGnore J. P. Konig S. Fales H. M. Keng Y. F. Zhang Z. Y. (1999). Regulation of PTP1B via glutathionylation of the active site cysteine 215. Biochemistry (Mosc.) 38, 6699–6705 10.1021/bi990240v 10350489

[B5] Benfeitas R. Selvaggio G. Antunes F. Coelho P. M. B. M. Salvador A. (2014). Hydrogen peroxide metabolism and sensing in human erythrocytes: a validated kinetic model and reappraisal of the role of peroxiredoxin II. Free Radic. Biol. Med. 74, 35–49 10.1016/j.freeradbiomed.2014.06.007 24952139

[B6] Bienert G. P. Møller A. L. B. Kristiansen K. A. Schulz A. Møller I. M. Schjoerring J. K. (2007). Specific aquaporins facilitate the diffusion of hydrogen peroxide across membranes. J. Biol. Chem. 282, 1183–1192 10.1074/jbc.M603761200 17105724

[B7] Branco M. R. Marinho H. S. Cyrne L. Antunes F. (2004). Decrease of H_2_O_2_ plasma membrane permeability during adaptation to H_2_O_2_ in *Saccharomyces cerevisiae*. J. Biol. Chem. 279, 6501–6506 10.1074/jbc.M311818200 14645222

[B8] Brigelius-Flohé R. Flohé L. (2011). Basic principles and emerging concepts in the redox control of transcription factors. Antioxid. Redox Signal. 15, 2335–2381 10.1089/ars.2010.3534 21194351 PMC3166203

[B9] Buettner G. R. Wagner B. A. Rodgers V. G. J. (2013). Quantitative redox biology: an approach to understanding the role of reactive species in defining the cellular redox environment. Cell Biochem. Biophys. 67, 477–483 10.1007/s12013-011-9320-3 22161621 PMC3661692

[B10] Chen C.-Y. Willard D. Rudolph J. (2009). Redox regulation of SH2-domain-containing protein tyrosine phosphatases by two backdoor cysteines. Biochemistry (Mosc.) 48, 1399–1409 10.1021/bi801973z 19166311

[B11] Chen K. Kirber M. T. Xiao H. Yang Y. Keaney J. F. (2008). Regulation of ROS signal transduction by NADPH oxidase 4 localization. J. Cell Biol. 181, 1129–1139 10.1083/jcb.200709049 18573911 PMC2442210

[B12] Covas G. Marinho H. S. Cyrne L. Antunes F. (2013). Activation of Nrf2 by H2O2: *de novo* synthesis versus nuclear translocation. Methods Enzymol. 528, 157–171 10.1016/B978-0-12-405881-1.00009-4 23849864

[B13] Cyrne L. Oliveira-Marques V. Marinho H. S. Antunes F. (2013). H_2_O_2_ in the induction of NF-κ B-dependent selective gene expression. Methods Enzymol. 528, 173–188 10.1016/B978-0-12-405881-1.00010-0 23849865

[B14] Dagnell M. Frijhoff J. Pader I. Augsten M. Boivin B. Xu J. (2013). Selective activation of oxidized PTP1B by the thioredoxin system modulates PDGF-? receptor tyrosine kinase signaling. Proc. Natl. Acad. Sci. U.S.A. 110, 13398–13403 10.1073/pnas.1302891110 23901112 PMC3746926

[B15] Denu J. M. Tanner K. G. (1998). Specific and reversible inactivation of protein tyrosine phosphatases by hydrogen peroxide: evidence for a sulfenic acid intermediate and implications for redox regulation. Biochemistry (Mosc.) 37, 5633–5642 10.1021/bi973035t 9548949

[B16] De Oliveira-Marques V. Cyrne L. Marinho H. Antunes F. (2007). A quantitative study of NF-kappa B activation byH_2_O_2_: relevance in inflammation and synergy with TNF-alpha. J. Immunol. 178, 3893–3902 10.4049/jimmunol.178.6.3893 17339489

[B17] Ferrer-Sueta G. Manta B. Botti H. Radi R. Trujillo M. Denicola A. (2011). Factors affecting protein thiol reactivity and specificity in peroxide reduction. Chem. Res. Toxicol. 24, 434–450 10.1021/tx100413v 21391663

[B18] Fisher-Wellman K. H. Neufer P. D. (2012). Linking mitochondrial bioenergetics to insulin resistance via redox biology. Trends Endocrinol. Metab. 23, 142–153 10.1016/j.tem.2011.12.008 22305519 PMC3313496

[B19] Flohe L. (1979). Glutathione peroxidase: fact and fiction. Ciba Found. Symp. 65, 95–122 383423

[B20] Forman H. J. (2007). Use and abuse of exogenous H_2_O_2_ in studies of signal transduction. Free Radic. Biol. Med. 42, 926–932 10.1016/j.freeradbiomed.2007.01.011 17349920 PMC1945171

[B21] Forman H. J. Maiorino M. Ursini F. (2010). Signaling functions of reactive oxygen species. Biochemistry (Mosc.) 49, 835–842 10.1021/bi9020378 20050630 PMC4226395

[B22] Forstrom J. W. Tappel A. L. (1979). Donor substrate specificity and thiol reduction of glutathione disulfide peroxidase. J. Biol. Chem. 254, 2888–2891 429326

[B23] Haque A. Andersen J. N. Salmeen A. Barford D. Tonks N. K. (2011). Conformation-sensing antibodies stabilize the oxidized form of PTP1B and inhibit its phosphatase activity. Cell 147, 185–198 10.1016/j.cell.2011.08.036 21962515 PMC3200309

[B24] Huang B. K. Sikes H. D. (2014). Quantifying intracellular hydrogen peroxide perturbations in terms of concentration. Redox Biol. 2, 955–962 10.1016/j.redox.2014.08.001 PMC4215397 25460730

[B25] Irani K. Xia Y. Zweier J. L. Sollott S. J. Der C. J. Fearon E. R. (1997). Mitogenic signaling mediated by oxidatnts in ras-transformed fibroblasts. Science 275, 1649–1652 10.1126/science.275.5306.1649 9054359

[B26] Iwakami S. Misu H. Takeda T. Sugimori M. Matsugo S. Kaneko S. (2011). Concentration-dependent dual effects of hydrogen peroxide on insulin signal transduction in H4IIEC hepatocytes. PLoS ONE 6:e27401 10.1371/journal.pone.0027401 22102892 PMC3216925

[B27] LaButti J. Chowdhury G. Reilly T. J. Gates K. S. (2007). Redox regulation of protein tyrosine phosphatase 1B (PTP1B) by peroxymonophosphate (= O3POOH). J. Am. Chem. Soc. 129:5320 10.1021/ja070194j 17411049 PMC2812892

[B28] Lee S. R. Kwon K. S. Kim S. R. Rhee S. G. (1998). Reversible inactivation of protein-tyrosine phosphatase 1B in A431 cells stimulated with epidermal growth factor. J. Biol. Chem. 273, 15366–15372 10.1074/jbc.273.25.15366 9624118

[B29] Le Moan N. Clement G. Le Maout S. Tacnet F. Toledano M. B. (2006). The *Saccharomyces cerevisiae* proteome of oxidized protein thiols: contrasted functions for the thioredoxin and glutathione pathways. J. Biol. Chem. 281, 10420–10430 10.1074/jbc.M513346200 16418165

[B30] Mahadev K. Zilbering A. Zhu L. Goldstein B. J. (2001). Insulin-stimulated hydrogen peroxide reversibly inhibits protein-tyrosine phosphatase 1b *in vivo* and enhances the early insulin action cascade. J. Biol. Chem. 276, 21938–21942 10.1074/jbc.C100109200 11297536

[B31] Makino N. Sasaki K. Hashida K. Sakakura Y. (2004). A metabolic model describing the H2O2 elimination by mammalian cells including H2O2 permeation through cytoplasmic and peroxisomal membranes: comparison with experimental data. Biochim. Biophys. Acta 1673, 149–159 10.1016/j.bbagen.2004.04.011 15279886

[B32] Marinho H. S. Cyrne L. Cadenas E. Antunes F. (2013a). H2O2 delivery to cells: steady-state versus bolus addition. Methods Enzymol. 526, 159–173 10.1016/B978-0-12-405883-5.00010-7 23791100

[B33] Marinho H. S. Cyrne L. Cadenas E. Antunes F. (2013b). The cellular steady-state of H_2_O_2_: latency concepts and gradients. Methods Enzymol. 527, 3–19 10.1016/B978-0-12-405882-8.00001-5 23830623

[B34] Marinho H. S. Real C. Cyrne L. Soares H. Antunes F. (2014). Hydrogen peroxide sensing, signaling and regulation of transcription factors. Redox Biol. 2, 535–562 10.1016/j.redox.2014.02.006 24634836 PMC3953959

[B35] Martínez-Acedo P. Núñez E. Gómez F. J. S. Moreno M. Ramos E. Izquierdo-Álvarez A. (2012). A novel strategy for global analysis of the dynamic thiol redox proteome. Mol. Cell. Proteomics 11, 800–813 10.1074/mcp.M111.016469 22647871 PMC3434769

[B36] Meng T.-C. Fukada T. Tonks N. K. (2002). Reversible oxidation and inactivation of protein tyrosine phosphatases *in vivo*. Mol. Cell 9, 387–399 10.1016/S1097-2765(02)00445-8 11864611

[B37] Miller E. W. Dickinson B. C. Chang C. J. (2010). Aquaporin-3 mediates hydrogen peroxide uptake to regulate downstream intracellular signaling. Proc. Natl. Acad. Sci. U.S.A. 107, 15681–15686 10.1073/pnas.1005776107 20724658 PMC2936599

[B38] Mishina N. M. Tyurin-Kuzmin P. A. Markvicheva K. N. Vorotnikov A. V. Tkachuk V. A. Laketa V. (2011). Does cellular hydrogen peroxide diffuse or act locally? Antioxid. Redox Signal. 14, 1–7 10.1089/ars.2010.3539 20690882

[B39] Oakley F. D. Abbott D. Li Q. Engelhardt J. F. (2009). Signaling components of redox active endosomes: the redoxosomes. Antioxid. Redox Signal. 11, 1313–1333 10.1089/ARS.2008.2363 19072143 PMC2842130

[B40] Oliveira-Marques V. Silva T. Cunha F. Covas G. Marinho H. S. Antunes F. (2013). A quantitative study of the cell-type specific modulation of c-Rel by hydrogen peroxide and TNF-? Redox Biol. 1, 347–352 10.1016/j.redox.2013.05.004 24024170 PMC3757704

[B41] Parsons Z. D. Gates K. S. (2013). Thiol-dependent recovery of catalytic activity from oxidized protein tyrosine phosphatases. Biochemistry (Mosc.) 52, 6412–6423 10.1021/bi400451m 23957891 PMC4006132

[B42] Paulsen C. E. Truong T. H. Garcia F. J. Homann A. Gupta V. Leonard S. E. (2012). Peroxide-dependent sulfenylation of the EGFR catalytic site enhances kinase activity. Nat. Chem. Biol. 8, 57–64 10.1038/nchembio.736 22158416 PMC3528018

[B43] Rasband W. (1997). ImageJ, U. S. National Institutes of Health. Bethesda, MD. Available online at: http://rsb.info.nih.gov/ij/, 1997–2006

[B44] Rawat S. J. Creasy C. L. Peterson J. R. Chernoff J. (2013). The tumor suppressor Mst1 promotes changes in the cellular redox state by phosphorylation and inactivation of peroxiredoxin-1 protein. J. Biol. Chem. 288, 8762–8771 10.1074/jbc.M112.414524 23386615 PMC3605693

[B45] Rinna A. Torres M. Forman H. J. (2006). Stimulation of the alveolar macrophage respiratory burst by ADP causes selective glutathionylation of protein tyrosine phosphatase 1B. Free Radic. Biol. Med. 41, 86–91 10.1016/j.freeradbiomed.2006.03.010 16781456 PMC2696202

[B46] Sies H. (2014). Role of metabolic H2O2 generation: redox signalling and oxidative stress. J. Biol. Chem. 289, 8735–8741 10.1074/jbc.R113.544635 24515117 PMC3979367

[B47] Tanner J. J. Parsons Z. D. Cummings A. H. Zhou H. Gates K. S. (2011). Redox regulation of protein tyrosine phosphatases: structural and chemical aspects. Antioxid. Redox Signal. 15, 77–97 10.1089/ars.2010.3611 20919935

[B48] Trindade D. F. Cerchiaro G. Augusto O. (2006). A role for peroxymonocarbonate in the stimulation of biothiol peroxidation by the bicarbonate/carbon dioxide pair. Chem. Res. Toxicol. 19, 1475–1482 10.1021/tx060146x 17112235

[B49] Tschopp J. Schroder K. (2010). NLRP3 inflammasome activation: the convergence of multiple signalling pathways on ROS production? Nat. Rev. Immunol. 10, 210–215 10.1038/nri2725 20168318

[B50] Van Eunen K. Bouwman J. Daran-Lapujade P. Postmus J. Canelas A. B. Mensonides F. I. C. (2010). Measuring enzyme activities under standardized *in vivo*-like conditions for systems biology. FEBS J. 277, 749–760 10.1111/j.1742-4658.2009.07524.x 20067525

[B51] Van Eunen K. Kiewiet J. A. L. Westerhoff H. V. Bakker B. M. (2012). Testing biochemistry revisited: how *in vivo* metabolism can be understood from *in vitro* enzyme kinetics. PLoS Comput Biol 8:e1002483 10.1371/journal.pcbi.1002483 22570597 PMC3343101

[B52] Voit E. (1991). Canonical Nonlinear Modeling: S-System Approach to Understanding Complexity. New York, NY: Van Nostrand Reinhold

[B53] Winterbourn C. C. Hampton M. B. (2008). Thiol chemistry and specificity in redox signaling. Free Radic. Biol. Med. 45, 549–561 10.1016/j.freeradbiomed.2008.05.004 18544350

[B54] Woo H. A. Yim S. H. Shin D. H. Kang D. Yu D.-Y. Rhee S. G. (2010). Inactivation of peroxiredoxin i by phosphorylation allows localized H2O2 accumulation for cell signaling. Cell 140, 517–528 10.1016/j.cell.2010.01.009 20178744

[B55] Zhou H. Singh H. Parsons Z. D. Lewis S. M. Bhattacharya S. Seiner D. R. (2011). The Biological Buffer Bicarbonate/CO _2_ Potentiates H_2_O_2_ -Mediated Inactivation of Protein Tyrosine Phosphatases. J. Am. Chem. Soc. 133, 15803–15805 10.1021/ja2077137 21913686 PMC3268130

